# *Orientus ishidae* (Hemiptera: Cicadellidae): Biology, Direct Damage and Preliminary Studies on Apple Proliferation Infection in Apple Orchard

**DOI:** 10.3390/insects14030246

**Published:** 2023-03-02

**Authors:** Giovanni Dalmaso, Claudio Ioriatti, Valeria Gualandri, Livia Zapponi, Valerio Mazzoni, Nicola Mori, Mario Baldessari

**Affiliations:** 1 Department of Biotechnology, University of Verona, Strada Le Grazie 15, 37134 Verona, Italy; 2 Centre for Technology Transfer, Fondazione Edmund Mach, Via E. Mach 1, 38010 San Michele all’Adige, Italy; 3 National Research Council of Italy, Institute of BioEconomy, 38098 San Michele all’Adige, Italy; 4 Research and Innovation Centre, Fondazione Edmund Mach, Via E. Mach 1, 38010 San Michele all’Adige, Italy

**Keywords:** leafhoppers, alien species, leaf depigmentation, phytoplasma vector, Apple Proliferation

## Abstract

**Simple Summary:**

A recent outbreak of the mosaic leafhopper (MLH), *Orientus ishidae*, in an apple orchard in Trentino made it urgent to assess the actual capability of this insect to cause damage to this crop. During the biennium from 2020–21, experiments were conducted both in field and semi-field conditions to ascertain whether MLH can (1) Complete the entire life cycle on cultivated apple trees; (2) Cause direct damage by feeding; and (3) Acquire the phytoplasma responsible of the Apple Proliferation (AP) disease. Our results proved that apple trees are suitable host plants for MLH, and that severe direct foliar damage is associated to its trophic activity. Such damage consists of diffuse chlorosis followed by necrosis that eventually take to leaf fall. The ability of MLH to acquire AP was confirmed in field trials. Our conclusions are that MLH is potentially a new pest of apple, although its role as a vector of AP must be confirmed with transmission bioassays. It will also be important to monitor this species in the apple orchards of other regions to prevent future outbreaks and consequent damage to production.

**Abstract:**

The mosaic leafhopper, *Orientus ishidae* (Matsumura), is an Asian species widespread in Europe that can cause leaf damage in wild trees and transmit disease phytoplasmas to grapevines. Following an *O. ishidae* outbreak reported in 2019 in an apple orchard in northern Italy, the biology and damage caused by this species to apples were investigated during 2020 and 2021. Our studies included observations on the *O. ishidae* life cycle, leaf symptoms associated to its trophic activity, and its capability to acquire “*Candidatus* Phytoplasma mali,” a causal agent of Apple Proliferation (AP). The results indicate that *O. ishidae* can complete the life cycle on apple trees. Nymphs emerged between May and June, and adults were present from early July to late October, with the peak of flight between July and early August. Semi-field observations allowed for an accurate description of leaf symptoms that appeared as a distinct yellowing after a one-day exposure. In field experiments, 23% of the leaves were found damaged. In addition, 16–18% of the collected leafhoppers were found carrying AP phytoplasma. We conclude that *O. ishidae* has the potential to be a new apple tree pest. However, further studies are required to better understand the economic impact of the infestations.

## 1. Introduction

*Orientus ishidae* (Matsumura), or “Mosaic Leafhopper” (MLH), is an East Palearctic species introduced first into the U.S. with the import of ornamental plant species [1,2] and, more recently, in Europe [3,4]. MLH has been collected from numerous broadleaf trees, such as *Quercus palustris* [5], *Betula* spp. [6,7], *Salix* spp. [3,6,8], *Corylus avellana*, *Ostrya carpinifolia*, *Carpinus betulus* [9], and, occasionally, also from herbaceous species like *Urtica dioica* [6] and from wild apple trees both in the USA [2] and in Europe [6,9,10]. MLH presence was also recorded in cultivated apple orchards: juveniles and adults in 1952 in the USA [11], and adults in 2016 in Slovenia [12]. However, in both situations, no further studies related to its biology and phenology were conducted. Finally, MLH presence has been recorded on grapevines (*Vitis vinifera*) in northern Italy [13], Slovenia [14], and Switzerland [15].

MLH is univoltine and overwinters as an egg under the bark of different trees (e.g., *Aralia* spp., *Alnus glutinosa*, *Fraxinus* spp., *C. avellana*, *Crataegus* spp., *C. betulus*, *Tillia mentosa*, *Ulmus minor*, *Robinia* sp., *V. vinifera*) [16,17,18]. Egg hatching from grapevines and wild plants begins in late spring in Northwestern Italy, the first instar nymphs occur from mid-May to early June, while the last nymphs (fifth instar) are present until early August [9,18,19]. Adults are active from the beginning of July until mid-October, with a flight peak between mid-July and early August [9,19,20]. MLH is associated with leaf damage symptoms, such as yellowing, necrosis, and stunted crown growth, in wild trees [2,16]. Furthermore, the leafhopper can transmit phytoplasmas such as “*Candidatus* Phytoplasma pruni,” the causal agent of peach-X disease [21,22], and, in laboratory trials, Flavescence dorée phytoplasma of Elm Yellows group 16SrV [9].

In 2019, a high MLH infestation was reported in apple orchards in Trentino (Italy), where plants showed symptoms of leaf depigmentation and necrosis in association with diffuse phyllotopsis [23]. Additionally, in the framework of an auchenorrhyncha biodiversity survey in the apple orchards of Trentino (Italy) carried out in 2014, few MLH individuals were collected for the first time in the region, and one of them tested positive for “*Candidatus* Phytoplasma mali,” a causal agent of the Apple Proliferation (AP) disease [10]. The “*Ca.* P. mali” is a quarantine phytoplasma widespread in Europe [24], causing severe symptoms on plants, such as witches’ brooms, and the production of small fruits with low organoleptic qualities [25]. The phytoplasma is transmitted by insect vectors, mainly by psyllids *Cacopsylla picta* (Foerster) (Hemiptera: Psyllidae), *Cacopsylla melanoneura* (Foerster), and, occasionally, by the leafhopper, *Fieberiella florii* (Stål) (Hemiptera: Cicadellidae) [26,27,28].

This work aimed to assess whether MLH can complete its full life cycle on apple trees. We also investigated the ability of MLH to cause direct feeding damage to the apple leaves and the capacity to acquire “*Ca*. P. mali”. The final aim was to define the specific interactions between MLH and apple trees to understand whether we are dealing with a potential new apple tree pest.

## 2. Materials and Methods

### 2.1. Apple Orchards Description

All trials were carried out at the fruit experimental station of Fondazione de Bellat in Valsugana, an apple-growing area in Trentino–Alto Adige region, Northeastern Italy (46°04′34.4″ N–11°47′19.3″ E). The studies were conducted in a 0.5 ha plot of apple trees, cv Golden Delicious on M9 rootstock, with 2.8% of symptomatic AP plants. The trees were planted in 1999 and maintained in the Spindel training form. No insecticide treatments were applied from May to the leaf fall. Other MHL individuals were collected from 0.8 ha of an apple orchard cv Reinette on M9 rootstock, planted in 2004, maintained organically, and located in Mollaro (Val di Non, Italy, 46°17′17.9″ N 11°03′57.0″ E) with 2.7% of AP symptomatic plants to detect if they were carrying “*Ca*. P. mali.”

### 2.2. Oviposition Site and Hatching Dynamics

To assess the MLH ability to complete its full life cycle on apple trees, we ascertained whether it could lay eggs on apple trees in natural conditions. For this purpose, 2–3-year-old apple branches (i.e., those having a bark layer that is the favorite substrate of MLH oviposition [18]) were collected in spring 2020 and 2021 from de Bellat site. The wood was cut into several pieces (15–20 cm each) and put inside plastic boxes (32 cm × 18.5 cm × 9.5 cm in 2020, 50 cm × 30 cm × 30 cm in 2021) closed by a plastic lid with two holes (2 cm × 5 cm) covered with anti-insect net. To preserve humidity, a layer of vermiculite (5 cm) was placed on the bottom of the boxes, and the canes were periodically sprinkled with water. To provide food for newly hatched nymphs, each box was supplied with a hazelnut leaf dipped in water, which was changed every two days. In 2020, each box was filled with 400–500 g of apple canes (total weight of the wood: 3.4 kg) and, in 2021, with 2 kg of canes (total weight 30 kg), which were stored in a greenhouse with controlled temperature (22 ± 1 °C). The boxes were inspected daily, and the emerged nymphs were transferred into a separate insect-proof cage containing an apple seedling. The experiment ended when hatching was not observed for a week.

### 2.3. Field Phenology and Flight Activities

During both 2020 and 2021 seasons, the presence of MLH nymphs and adults were visually inspected on apple trees from May to November to study its phenology. Three trees were randomly chosen weekly from each row (n rows = 8) at the de Bellat site; all the leaves from two randomly chosen branches per tree (i.e., south and north exposition) were surveyed in 2020 and 2021 (total n of surveyed branches/week = 48). The number of I–II nymph instars (N1–N2 winglets absent), III–V nymph instars (N3–N5, winglets present), and adults was counted.

Three yellow sticky traps (25 cm × 10 cm, Glutor, CBC (Europe) S.r.l. BIOGARD DIVISION, 24050 Grassobbio (BG), Italy) were attached in the orchard to monitor the abundance, flight activity, and sex ratio of adults [29]. The traps were secured to the middle support wire at 1.90 m from the ground and were replaced weekly from the end of June to the beginning of November.

### 2.4. Damage Characterisation and Quantification

Two sets of observations were carried out in 2020 to evaluate the MLH damage to apples: one in semi-field conditions, on 3-year-old potted plants, and a second one in the open field. In the semi-field trial, 10 MLH nymphs (N4–N5) were placed inside a plastic sleeve cage (40 cm × 20 cm nylon sleeve, BugDorm, Megaview Science Co., Ltd., Taichung 407008, Taiwan) positioned around a branch of the apple trees (one sleeve/plant). The plants were kept under a polytunnel to protect them from wind and rain. Each sleeve contained 10 leaves of similar size. The leaves in individual cages were subjected to three different treatments named according to the time of permanence of the MLH in the cage: 24 h, 72 h, and N h. In the latter, the insects were kept inside the sleeves until death. In the control treatment, no insects were placed into the sleeve. The experiment started on 6 July, and each treatment was replicated five times. Immediately after the removal of the insects (using a mouth aspirator), the number of leaves showing symptoms compatible with the MLH injuries was counted. From then on, the damage assessment was conducted weekly and lasted for 7 weeks. The leaf symptoms were divided into five classes of severity: Class 0 (Cl0), no evident symptoms; Class 1 (Cl1), chlorosis occurring in a leaf portion; Class 2 (Cl2), diffuse chlorosis and necrosis appearance; Class 3 (Cl3), necrosis extended to most of the surface; and Class 4 (Cl4), necrosis all over the leaf area in conditions of pre-phyllotopsis or leaf fall. To quantify the symptoms and make comparisons across the different treatments, an index of damage (ID) was created with the formula:

$$\mathrm{ID}=\left(\frac{\left(0\times n\mathrm{Cl}0+1\times n\mathrm{Cl}1+2\times n\mathrm{Cl}2+3\times n\mathrm{Cl}3+4\times n\mathrm{Cl}4\right)}{4N}\right)$$

where “*n*” is the number of leaves observed for each class and “*N*” the total number of leaves per treatment (*N* = 10). ID ranged from 0 (no symptoms) to 1 (Cl4 symptoms) on all sampled leaves.

In the field trials, leaf sampling was conducted weekly from the end of June to September 2020 at the de Bellat site to estimate the amount of damaged leaves in open-field condition. In addition, to evaluate the loss of photosynthetic area, the percentage of leaf damaged surface was assessed. For each observation, four apple trees were randomly chosen from each row (eight total rows, *n* = 32) and 25 leaves per plant were inspected at a height of about 1.80 m from the ground.

### 2.5. AP Phytoplasma Infection

In order to assess the AP phytoplasma infection rate in MLH, adults were collected at the de Bellat site and in the Mollaro orchard using a mouth aspirator from AP-symptomatic trees (i.e., showing witches’ brooms and enlarged/dentate stipules) and stored at −20 °C until molecular analyses. To detect the presence of “*Ca*. P. mali,” the total genomic DNA was extracted from single specimens using the commercial kit NucleoSpin Tissue (Macherey-Nagel GmbH and Co., KG, 52355 Düren, Germany). The collected insects were subjected to PCR amplification with two primers: fAT (forward, 5′ CAT CAT TTA GTT GGG CAC TT-3′) and rAT (reverse, 5′–GGC CCC GGA CCA TTA TTT ATT–3′) that amplify in the region 16S/23S [30]. The PCR assays were performed with 2 mL of DNA, 10 mL of Master Mix Green Promega, 0.75 mL of primer fAT, 0.75 mL of primer rAT, and 0.75 mL of sterile water for a final reaction volume of 20 μL. The PCR parameters were as follows: an initial denaturation of 2 min at 95 °C, and 35 cycles with 60 s at 95 °C, 45 s at 57 °C, 60 s at 72 °C, and a final extension of 5 min at 72 °C. PCR products were analyzed by electrophoresis on 1.5% *w*/*v* agarose gels stained with Ethidium bromide and visualized under UV light (ChemiDoc XRS, BioRad, 94547 Hercules, CA, USA).

### 2.6. Data Analysis

Since ID value ranged in the unit interval (0–1), the effect of the treatment and week on damage was analyzed with a beta regression with a “loglog” link. The beta regression was conducted in R [31] using the betareg package [32] considering the independent effect of the explanatory variables and their interaction. Model assumptions were verified by plotting residuals against fitted values and against each covariate in the model. The candidate models were compared with a likelihood-ratio test. Predictor effect plots [33] were used to visualize the fitted coefficients.

## 3. Results

### 3.1. Oviposition Site and Hatching Dynamics

**Figure 1:**
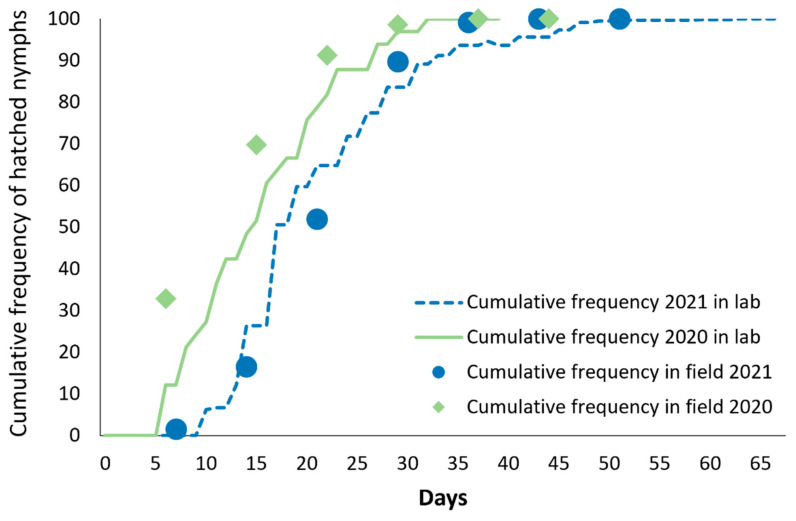
Comparison between cumulative frequency distribution of *O. ishidae* egg-hatching obtained from apple wood in lab (line 2020 vs. dotted line 2021) and in field (rhombus 2020 vs. circle 2021).

In 2020, 33 MLH juveniles emerged from the stored canes in the laboratory with a density of 9.71 nymphs/kg of wood. In lab conditions, the hatching period lasted 27 days and 79% of the individuals emerged in the first two weeks. In the field, the MLH first instars were observed on 3 June and were found during the next 31 days with 91% of them within the first 16 days (Figure 1).

In 2021, 950 MLH juveniles emerged from the stored canes in the greenhouse with a density of 30.2 nymphs/kg of wood. The hatching period lasted 53 days from 3 May to 25 June, and 91% of them occurred within 23 days from the first hatch (Figure 1). In the field sampling, the MLH first instars were detected between 18 May and 23 June, with 89% of them hatched within 15 days of first detection (Figure 1).

### 3.2. Field Phenology

**Figure 2:**
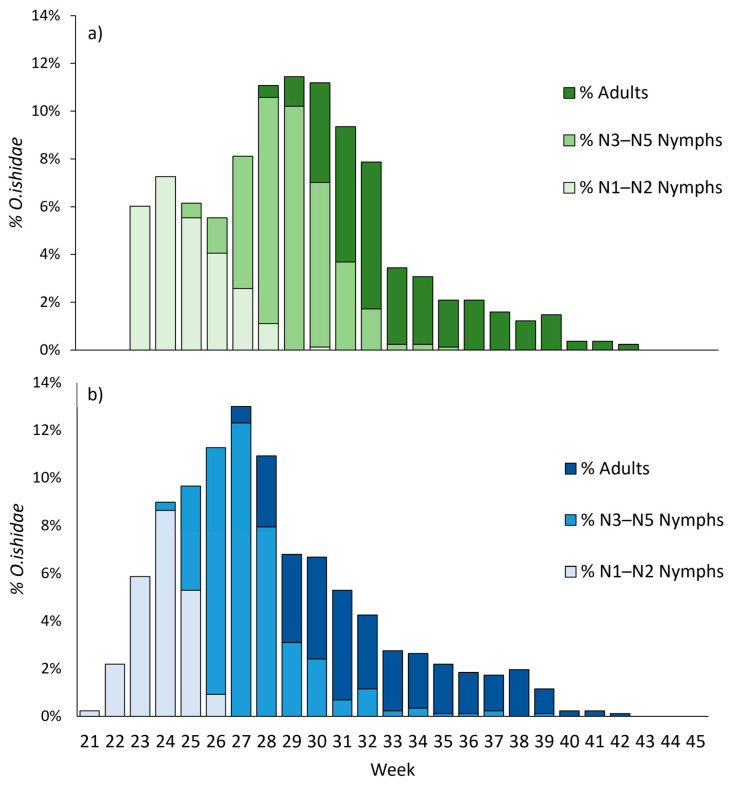
Seasonal abundance of *O. ishidae*, expressed as percentage of total *O. ishidae* finding in the season. Juvenile instars and adults in 2020 (**a**) (total *O. ishidae* = 813) and 2021 (**b**) (total *O. ishidae* = 869).

In the de Bellat site, a total of 813 and 869 MLH individuals were sampled in 2020 and 2021, respectively. As a whole, the population dynamic was similar between the two years, although we observed a 1–2-week anticipation in 2021 (Figure 2). The first individuals were observed at the beginning of June and from mid-May in 2020 and 2021, respectively. Nymphs (N3–N5) were observed starting from Week 25 in 2020 and from Week 24 in 2021. Accordingly, in 2021, the first adults were observed 1 week earlier than in 2020 (Figure 2). The higher population density (mobile forms) was observed after the disappearance of N1–N2, both in 2020 and 2021, when the N3–N5 were dominant and at the appearance of the first adults. The last juveniles were found in late August 2020 and mid-September 2021, suggesting that there were still eggs hatching in late July or early August.

N1 and N2 were mostly found on the lower surface of the leaves and showed relatively low mobility. In contrast, N3–N5 and adults were mainly observed on the upper leaf surface and jumped/flew away when disturbed.

### 3.3. Flight Activities

**Figure 3:**
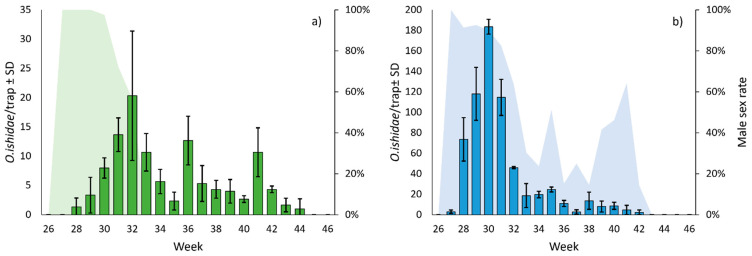
Seasonal trend of *O. ishidae* adults captured by yellow sticky traps (bar plot with standard deviation) in 2020 (**a**) and 2021 (**b**) placed inside the apple orchards (*n* = 3). The male sex rate is also showed (area plot).

In 2020, the captures with sticky traps started from 11 July, the flight peak was observed at the beginning of August, and adults were detected until the end of October. Males were caught for 3 weeks after the beginning of the flight, with the peak flight recorded early in August, and no catches were recorded from mid-August onwards. The first females were found at the end of July, with the peak flight observed early in September, and no captures were recorded from late October onwards (Figure 3).

In 2021, the captures started from 2 July, the flight peak was recorded in mid-July and no adults were captured from mid-October onwards. In 2021, the captures were, on average, 6 times higher than those recorded in 2020 (Figure 3). In 2021, only males were recorded during the first week, with the peak male flight occurring in mid-July and, unlike 2020, males did not disappear completely after the peak flight and were observed until mid-October. The first females were found a week after the males appeared in July, with the female’s peak flight delayed by a week compared to that of males and no catches were recorded from mid-October onwards (Figure 3).

### 3.4. Damage Characterisation

**Figure 4:**
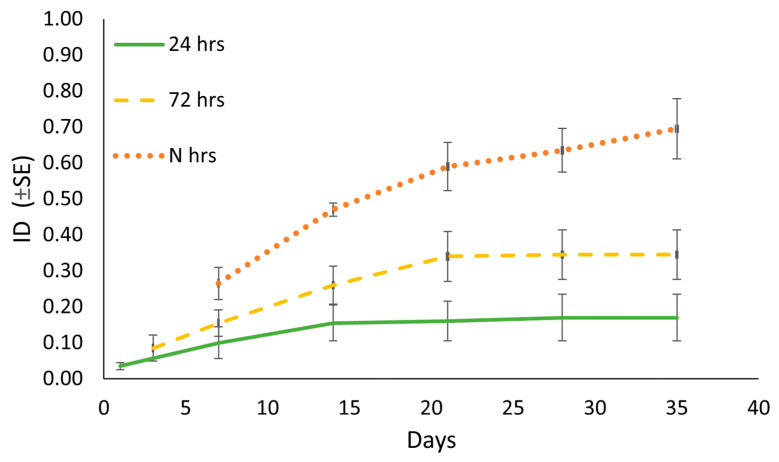
Mean indexed damage (ID), over the five weeks in the different treatments (*n* = 5).

In the semi-field trials (Figure 4), both “24 h” and “72 h” treatments were associated with a distinct leaf damage and low symptom severity (Cl1–Cl2) that was already evident when the insects were removed (ID24 h = 0.04; ID72 h = 0.09). After insect removal, the symptomatic area further extended (Cl3) for the following three weeks with a final value of ID = 0.17 and 0.35 for “24 h” and “72 h,” respectively. From that moment on, both the area and type of damage (as class) did not change, with a total of two fallen leaves recorded in the “24 h” and one in the “72 h.” The “N h” treatment showed the most severe damage, with the largest number of leaves that reached Cl3 (20%) and Cl4 (54%), and the symptom extension that increased until the end of the trials (ID = 0.66). No symptomatic leaves were found throughout the test period in the control.

**Figure 5:**
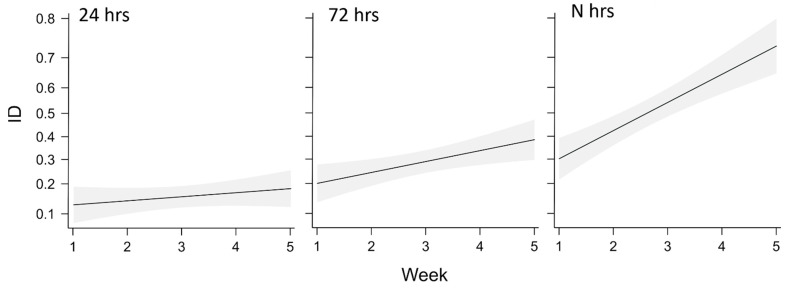
Effect plot for the variables included in the beta regression model, showing model fit and confidence intervals (shaded area).

Beta regression validation indicated no problems and, according to the likelihood ratio test, the model with the interaction (Treatment × Week) was significantly preferable (Χ^2^ = 1.109, df = 7, *p*-value < 0.001). The selected beta regression model confirmed the qualitative assessment, with a significant effect of the interaction between the “N h” and time (Week) (Table 1). While the interactions “24 h”—week and “72 h”—week were not significant according to the model, it showed a steep increase in terms of ID for “N h”—week (Figure 5).

**Figure 6:**
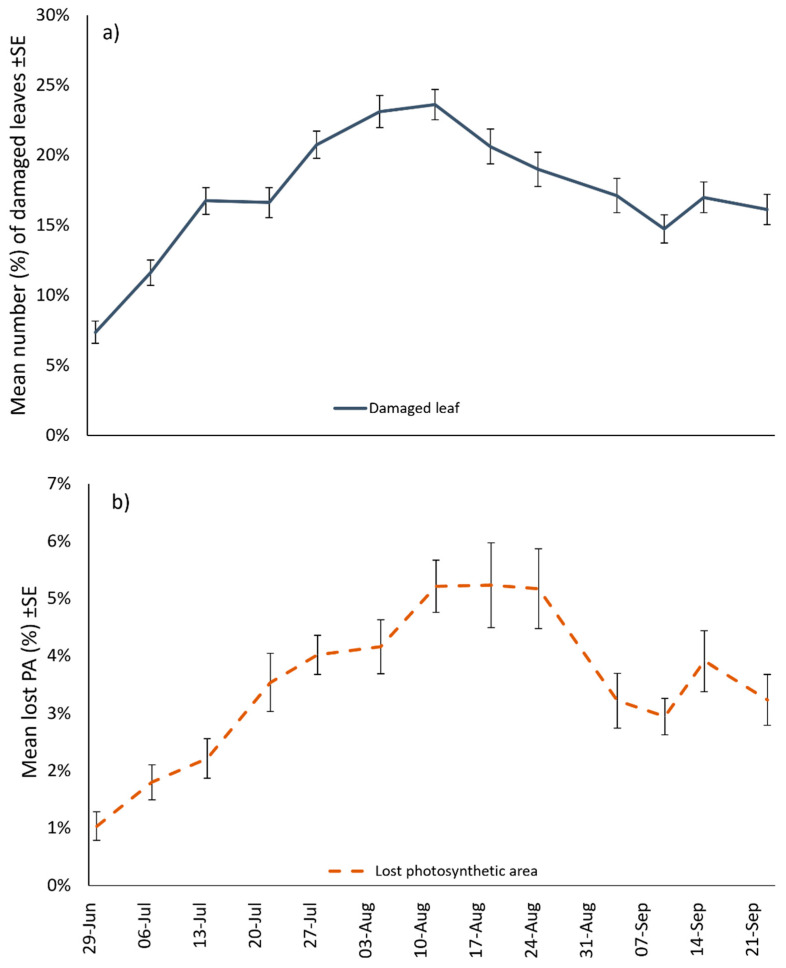
Seasonal trend of *O. ishidae* direct damaged in apple orchards, expressed (**a**) As mean percentage of affected leaves and (**b**) Mean percentage of lost photosynthetic area (PA) (*n* = 32).

In the open field plot (Figure 6), the percentage of leaves damaged increased from the end of June to mid-August, when the highest damage of 23% was observed (12 August). From the beginning of September until the end of the trial, leaf damage decreased, with values ranging between 15% and 17% of symptomatic leaves. The largest damaged leaf area was 5% of the total sampled leaf area and was recorded on 22 August. From that moment, the percentage of damaged leaf area decreased, reaching approximately 3% at the end of September.

### 3.5. AP Infectivity

A total of 119 insects were analyzed. Data showed a percentage of leafhoppers harboring “*Ca*. P. mali”-ranging from 15.6% in the Mollaro orchard to 18.6% in the de Bellat site. Similarly, the percentage of symptomatic plants was similar in the two orchards (2.8% and 2.7%) (Table 2).

## 4. Discussion

Our research demonstrated that MLH is able to (1) Complete the entire life cycle on cultivated apple trees; (2) Cause direct leaf damage by feeding; and (3) Harbor the phytoplasma associated with the Apple Proliferation (AP) disease.

Similar to what was observed on grapevines [18], MLH nymphs were obtained from eggs laid in 2–3-year-old branches, which, therefore, constitute a suitable site for oviposition. Interestingly, the level of oviposition in apple trees observed in 2020 (10 nymphs/kg) was more than 10 times higher than what was previously recorded from grapevine canes collected in organic vineyards (0.7 nymphs/kg) [18]. This data was surprisingly high considering that, between June and August 2019, three treatments with acetamiprid-based insecticide were applied in the same orchard to control the brown marmorated stink bug [34]. Although this insecticide application was not specific to control MLH, the application period and the broad spectrum of action [35] should have affected the population and, consequently, the oviposition level, as already hypothesized in IPM vineyards [9]. We cannot exclude that the high oviposition level recorded in 2020 was caused by the immigration of females from the wild vegetation in the surrounding areas, at the end of the summer when the orchard was no longer protected by insecticide treatment. However, research conducted in vineyards suggested that MLH adults do not move massively from the edges into the cultivated area [9]. In this regard, it could be possible that apple trees represent a more favorable host plant than grapevines, and for this reason they could attract more MLH adults from other neighboring plants. In addition, in 2021, the number of hatched nymphs tripled, reaching hatching levels even higher than those reported from wild plants (e.g., hornbeam, alder, *Robinia* spp. etc., [17]). Even though these results must be confirmed with additional observations in other years and areas, they nevertheless strongly suggest that apple trees are not only occasional hosts, infested according to the botanical context in which the orchard is inserted, but rather that they can be key host plants for MLH.

As for the MLH phenology, the observed dynamics of the different life stages matched the data of previous research, even though we found differences between the two years of investigation. The first appearance of nymphs was recorded early in June in 2020, as also reported by Valley and Wheeler Jr. on the ornamental honey locust (*Gleditsia triacanthos*) in Pennsylvania (USA) [16]. On the other hand, in 2021, first instars were detected in mid-May as previously reported in Piedmont (Italy) on grapevines and wild plants [9,19]. The observed discrepancy probably depended on a different hatching period due to climatic conditions (particularly temperature) to which overwintering eggs have been previously exposed. This phenomenon was demonstrated for other leafhoppers such as *Scaphoideus titanus* Ball, whose egg hatching period were susceptible to great variability depending on the heat treatments to which the overwintering eggs have been subjected [36,37,38].

As for the adults, their presence was observed from the beginning of July to the end of October in both years, with the peak of flight occurring between mid-July and the beginning of August. A similar flight trend was already reported in other studies conducted in the orchards of honey locust trees, vineyards, and wild plants (i.e., hornbeams, willows) [9,16,19,20].

Our results suggest that the population rose from 2020 to 2021. In particular, both hatching dynamics and sticky traps indicated considerably higher numbers of MLH in the second year of trials. On the contrary, we did not observe such a difference with the visual assessment method, which seemed to indicate similar values in the biennium. The three methods target different stages and, therefore, could be not accurate. For example, the hatch data show only the potential new population in the orchard and do not take into account the mortality of eggs or nymphs during juvenile development due both to abiotic and biotic factors (i.e., antagonists). On the other hand, chromotropic traps provide data only of the adult stage, and the catches are subject to different variables (e.g., exposure, location in canopy, weather, etc. [29]). Even in this case, abiotic factors can play a crucial role in determining the flight activity. This discrepancy with the visual assessment may be explained by the fact that we used as the sample unit the branch and not the number of leaves, which could be different for each branch in different years. Nevertheless, measuring the quantity of MLH in the orchard was out of the scope of this work, which aimed to study the MLH biology and phenology. Specific studies on the best sampling method will have to be conducted in the future, considering multiple variables such as the aggregate behavior on leaves/sprouts that MLH exhibited on wild species (e.g., hazelnut) [18]. In this case, a landscape analysis will be also crucial to define how the agroecosystem context can affect the MLH incidence on a crop and, in particular, on an apple orchard.

Sticky traps were useful to define the MLH flight behavior and the gender differences. The sex rate analysis showed a prevalence of males in the first part of the season (July) and a progressive inversion with a prevalence of females starting from August. This data is in accordance with observations conducted on other leafhoppers [39,40] and can be due to the different behavior of the sexes. In fact, MLH uses vibrational signals for mating communication, and males actively search for females by emitting species-specific vibrational signals [41]. A typical strategy of leafhoppers is the so-called “call-fly,” which consists of the emission of vibrational signals (i.e., calling signals) alternated with flights from leaf to leaf [42]. This is a method to increase the active space of signaling leafhoppers [43], and our study supports the use of call-fly strategy by MLH during the first part of the summer until the male peak. Then, starting from August, we can assume that most of the mating has been completed and, while males relatively quickly disappear, females begin to be more mobile to oviposit. These dynamics, therefore, seem to indicate the maximum oviposition period between August and early September, in correspondence to the female peak of captures.

As for the investigation about the feeding damage, our trial in controlled conditions indicates that symptoms appear pretty soon after the inoculation, but also that they further evolve for a period of 1–2 weeks from the insect removal. This opens two hypotheses about the nature of the leaf damage. Phytophagous hemipterans can be divided into two categories according to their feeding strategies [44]: sheath feeders and cell rupture feeders. Sheath feeders, such as aphids and most leafhoppers [45], produce a sheath of gel saliva along the stylet and inject water saliva containing digestive enzymes before feeding on the phloem or xylem. This strategy is associated to immediate leaf damage that does not evolve once the feeder is removed [45,46,47,48,49]. This group includes the most effective phytoplasma vectors [50]. Cell rupture feeders, on the other hand, do not make a complete salivary sheath. Instead, their stylets move in and out of the plant tissues, mechanically lacerating cells and simultaneously secreting watery saliva. This strategy is typical of members of the leafhoppers of the subfamily typhlocybinae and is associated with a specific leaf symptom called “hopperburn.” Insects with this strategy have a low vector capability [46,50,51]. Our experiments, therefore, suggest that MLH causes “hopperburn-type” symptoms, supporting the thesis that this species is a cell rupture feeder [9]. On the other hand, it cannot be excluded that the progression of the symptoms may be caused by the action of phytotoxic saliva, similar to what happens for some aphids (e.g., *Schizaphis graminum* (Rond.), *Diuraphis noxia* (Kurdjumov), which instead are typical sheath feeders [52,53,54,55]. Moreover, “hopperburn” symptoms were also reported from non-typhlocybinae auchenorrhyncha, such as delphacids *Nilaparvata lugens* (Stål) and *Sogatella furcifera* (Horvath) [56]. These two planthoppers are sheath feeders that can transmit viral disease but also can cause extensive direct damage to host plants [57,58,59] due to phytotoxic saliva [46]. The symptoms that we observed in the case of MLH seem to be similar to that observed in those planthoppers; in fact, the spread of saliva through the vascular system would explain the delayed appearance and extension of symptoms in apparently healthy leaves in the “24 h” and “72 h” after the insect removal, and that the phylloptosis of leaves is only partially symptomatic. In addition, the fact that, to date, no members of the subfamily deltocephalinae are known to be cell rupture feeders supports our hypothesis [46]. Further studies using specific techniques aiming at describing the MLH feeding behavior, e.g., electrical penetration graph (EPG), could clarify this significant aspect [60,61].

It is important to note that when MLH remained on the plant throughout the trial period (“N h”), we observed quite a remarkable difference with the other treatments in damage severity with 54% of the leaves showing full surface necrosis (Cl4), most of which had fallen by the end of the experiment. Such high damage was the result of MLH continuous trophic activity for a prolonged period, but we cannot exclude that specimens not enclosed in sleeves and free to move would eventually move to other leaves thus limiting the total phylloptosis.

Interestingly, field and semi-field trials yielded similar results. In fact, we observed MLH damage associated to a relevant percentage of apple leaves: We found a maximum of 23% of symptomatic leaves, with a total leaf surface reduction of 5%. Even though this value hardly affects the photosynthesizing capacity of the apple, and consequently the production, we did not account for the leaves fallen to the ground in our sampling, which was suggested by the observed reduction of percentage damaged leaves on the plants from August. In this regard, we can hypothesize that MLH nymphs, as in many other leafhoppers, cause more damage to the host plant than adults. For this reason, the symptom severity grows faster in the first part of the apple growing season. In this context, the observed reduction can be explained by phylloptosis, which would be higher than the appearance of new symptomatic leaves. However, the actual severity of the phylloptosis could be measured only by means of dedicated experiments with the aim to count the number of fallen leaves. This aspect deserves further investigation to better understand the potential direct damage caused by MLH to apple. In addition, the monitoring of MLH should be extended to other orchards with different varieties and to other geographical areas to assess whether the reported invasion in Trentino is an isolated case or is a clue of an incipient new problem.

Finally, regarding the association between MLH and AP phytoplasma, our molecular analysis revealed that 15–18% of tested individuals were positive to “*Ca*. P. mali”. Making a comparison between this data and the natural infection rate of *C. melanoneura* or *C picta* (i.e., the two main AP vectors) is difficult due to the infective rates of the psyllids, which vary considerably according to the geographic region and the development stage (e.g., overwintering, nymphs, emigrants) [62,63,64,65,66]. However, overwintering *C. melanoenura* were found with a natural infectivity range between 1% and 23% in Trentino [62,65,66], comparable with what we observed in MLH, whereas overwintering *C. picta* showed a higher natural infectivity reaching up to 45% of infected individuals [63]. The leafhopper *F. flori*, the third known AP vector, which is taxonomically more closely related to MLH, is characterized by lower infectivity values in apple orchards (on average 5.7%), even though when collected from wild vegetation, the percentage of AP infected individuals increased reaching values between 9.1 to 20%, comparable with MLH [28]. At any rate, the acquisition of phytoplasma by a phloem-feeding Auchenorrhyncha is not sufficient proof to state that the insect is a vector [67]. For example, “*Ca*. P. mali” was detected in many other phloem-feeding Hemiptera, such as the planthopper *Metcalfa pruinosa* (Say) [68] and the psyllids *Cacopsylla peregrina* (Förster) and *Ctenarytaina eucalypti* (Maskell) [69,70]. For all these species, transmission trials were not carried out, so the potential role of these insects in the AP epidemiology is still unknown. Other Hemiptera that successfully acquired “*Ca*. P. mali” then were not able to transmit it to other plants, as reported for different apple aphids (e.g., *Aphis pomi* (DeGeer) *Dysaphis plantaginea* (Passerini), *Eriosoma lanigerum* (Hausmann) [71], and leafhoppers (e.g., *Empoasca vitis* (Göethe) [72]. Thus, our experiments do not allow us to demonstrate whether MLH plays a role in the AP epidemiology. Nevertheless, they provide a basis for future experiments of transmission [73].

## 5. Conclusions

The present research unveiled several aspects related to the biology of *O. ishidae* and proved its interaction with apple trees. Many other aspects deserve to be more deeply investigated, in particular, the MLH role as a candidatus vector of the AP phytoplasma. The acquisition of this knowledge will be fundamental to define the importance of MLH in the apple production and its potential economic impact in the future.

## Figures and Tables

**Table 1:** Estimated regression parameters, z-values, and *p*-values for the beta regression. Pseudo R-squared: 0.658.

Treatments	Estimate	Std. Error	z Value	Pr (>|z|)
Treatment 72 h	0.157	0.215	0.729	0.466
Treatment N h	0.265	0.231	1.151	0.250
Week	0.048	0.045	1.074	0.283
Treatment 72 h: Week	0.082	0.066	1.241	0.215
Treatment N h: Week	0.288	0.075	3.826	>0.001
Intercept	−0.769	0.149	−5.147	>0.001

**Table 2:** Results of the molecular analysis of collected MLH in the two different sites.

Site	De Bellat	Mollaro
Data of collection	14 August 2020	3 September 2020
Collected MLH	43	76
AP-positive MLH	8	12
% AP-positive MLH	18.6	15.7
% Infected plants	2.8	2.7

## Data Availability

The data presented in this study are available on request from the corresponding author.

## References

[1] Sanders J.G., DeLong D.M. (1919). Eight New “Jassids” from the Eastern United States. Ann. Entomol. Soc. Am..

[2] Felt E.P., Bromley S.W. (1941). New and Unusual Shade Tree Pests. J. Econ. Entomol..

[3] Guglielmino A. (2005). Observations on the genus *Orientus* (Rhynchota Cicadomorpha Cicadellidae) and description of a new species: *O. amurensis* n. sp. from Russia (Amur Region and Maritime Territory) and China (Liaoning Province). Marburger. Entomologische. Publikationen..

[4] Blaser S.C. (2019). Invasion Genetics and Development of Rapid Diagnostics of Insect Pests on Traded Plants. Ph.D. Thesis.

[5] Johnson M., Freytag P. (2001). Leafhoppers (Homoptera: Cicadellidae) on pin oak in Kentucky. J. Kans. Entomol. Soc..

[6] Nickel H. (2010). First addendum to the Leafhoppers and Planthoppers of Germany (Hemiptera: Auchenorrhyncha). Cicadina.

[7] Koczor S., Bagarus A.K., Karap A.K., Varga Á., Orosz A. (2013). A Rapidly spreading potential pest, *Orientus ishidae* identified in Hungary. Bull. Insectology.

[8] Mazzoni V. (2005). Contribution to the knowledge of the Auchenorrhyncha (Hemiptera Fulgoromorpha and Cicadomorpha) of Tuscany (Italy). Redia.

[9] Lessio F., Picciau L., Gonella E., Mandrioli M., Tota F., Alma A. (2016). The Mosaic Leafhopper *Orientus ishidae*: Host Plants, Spatial Distribution, Infectivity, and Transmission of 16SrV Phytoplasmas to Vines. Bull. Insectology.

[10] Oppedisano T. (2017). New Insights into the Biology and Ecology of the Insect Vectors of Apple Proliferation for the Development of Sustainable Control Strategies. Ph.D. Thesis.

[11] Garman P., Townsend J.F. (1952). Control of Apple Insects: Bulletin 552.

[12] Lešnik M., Seljak G., Vajs S. Populacijska dinamika škržatka *Orientus ishidae* Matsumura v nasadih jablan v letih 2015 in 2016. Proceedings of the 13th Slovenian Conference on Plant Protection with International Participation.

[13] Gaffuri F., Sacchi S., Cavagna B. (2011). First Detection of the Mosaic Leafhopper, *Orientus ishidae*, in Northern Italian Vineyards Infected by the Flavescence Dorée Phytoplasma. New Dis. Rep..

[14] Mehle N., Seljak G., Rupar M., Ravnikar M., Dermastia M. (2010). The First Detection of a Phytoplasma from the 16SrV (Elm Yellows) Group in the Mosaic Leafhopper Orientus ishidae. New Dis. Rep..

[15] Trivellone V., Knop E., Turrini T., Andrey A., Humbert J.-Y., Kunz G. (2015). New and Remarkable Leafhoppers and Planthoppers (Hemiptera: Auchenorrhyncha) from Switzerland. Mitt. Schweiz. Entomol. Ges..

[16] Valley K.R., Wheeler A.G. (1985). Leafhoppers (Hemiptera: Cicadellidae) associated with ornamental honey locust: Seasonal history, habits, and descriptions of eggs and fifth instars. Ann. Entomol. Soc. Am..

[17] Desqué D., Salar P., Danet J.L., Lusseau T., Garcion C., Moreau E., Dubus C., Dureuil J., Delbac L., Binet D. (2019). Impact of *Orientus ishidae* on “Flavescence Dorée” emergence in the vineyards of riparian ecosystems. Phytopathog. Mollicutes.

[18] Lessio F., Bocca F., Alma A. (2019). Development, spatial distribution, and presence on grapevine of nymphs of *Orientus ishidae* (Hemiptera: Cicadellidae), a new vector of flavescence dorée phytoplasmas. J. Econ. Entomol..

[19] Parise G. (2017). Notes on the biology of *Orientus ishidae* (Matsumura, 1902) in Piedmont (Italy) (Hemiptera: Cicadellidae: Deltocephalinae). Cicadina.

[20] Casati P., Quaglino F., Tedeschi R., Spiga F.M., Alma A., Spadone P., Bianco P.A. (2010). Identification and molecular characterization of ‘*Candidatus* Phytoplasma mali’ Isolates in North-Western Italy. J. Phytopathol..

[21] Rosenberger D.A. (1978). Leafhopper vectors of the peach X-disease pathogen and its seasonal transmission from chokecherry. Phytopathology.

[22] Davis R.E., Zhao Y., Dally E.L., Lee I.M., Jomantiene R., Douglas S.M. (2013). “*Candidatus* Phytoplasma pruni”, A novel taxon associated with x-disease of stone fruits, *Prunus* spp.: Multilocus characterization based on 16S RRNA, SecY, and ribosomal protein genes. Int. J. Syst. Evol. Microbiol..

[23] Baldessari M., Dalmaso G., Mazzoni V., Mori N. (2020). Infestazioni su melo in Trentino di *Orientus ishidae*. Inf. Agrar..

[24] “Candidatus Phytoplasma mali” (PHYPMA)[Overview]|EPPO Global Database. https://gd.eppo.int/taxon/PHYPMA.

[25] Janik K., Barthel D., Oppedisano T., Anfora G. (2020). Apple Proliferation: A Joint Review.

[26] Frisinghelli C., Delaiti L., Grando M.S., Forti D., Vindimian M.E. (2000). *Cacopsylla Costalis* (Flor 1861), as a Vector of Apple Proliferation in Trentino. J. Phytopathol..

[27] Tedeschi R., Alma A. (2004). Transmission of apple proliferation phytoplasma by *Cacopsylla melanoneura* (Homoptera: Psyllidae). J. Econ. Entomol..

[28] Tedeschi R., Alma A. (2006). *Fieberiella florii* (Homoptera: Auchenorrhyncha) as a Vector of “*Candidatus* Phytoplasma mali”. Plant Dis..

[29] Pavan F., Cargnus E., Tacoli F., Zandigiacomo P. (2021). Standardization and criticism of sampling procedures using sticky card traps: Monitoring sap-sucking insect pests and *Anagrus atomus* inhabiting European vineyards. Bull. Insectology.

[30] Smart C.D., Schneider B., Blomquist Ç.L., Guerra L.J., Harrison N.A., Ahrens U., Lorenz E., Seemüller K.H., Kirkpatrick B.C. (1996). Phytoplasma-Specific PCR Primers Based on Sequences of the 16S-23S RRNA Spacer Region. Appl. Environ. Microbiol..

[31] R: A Language and Environment for Statistical Computing. R Foundation for Statistical Computing, Vienna, Austria|CiNii Research. https://cir.nii.ac.jp/crid/1574231874043578752.

[32] Cribari-Neto F., Zeileis A. (2010). Beta Regression in R. J. Stat. Softw..

[33] Fox J., Weisberg S. (2018). An R companion to Aapplied Regression.

[34] Baldessari M. (2022). Personal Communication.

[35] Elbert A., Haas M., Springer B., Thielert W., Nauen R. (2008). Applied Aspects of Neonicotinoid Uses in Crop Protection. Pest Manag. Sci..

[36] Chuche J., Thiéry D. (2009). Cold Winter Temperatures Condition the Egg-Hatching Dynamics of a Grape Disease Vector. Naturwissenschaften.

[37] Chuche J., Thiéry D. (2014). Biology and Ecology of the Flavescence Dorée Vector *Scaphoideus titanus*: A Review. Agron. Sustain. Dev..

[38] Falzoi S., Lessio F., Spanna F., Alma A. (2014). Influence of temperature on the embryonic and post-embryonic development of *Scaphoideus titanus* (Hemiptera: Cicadellidae), vector of grapevine Flavescence Dorée. Int. J. Pest Manag..

[39] Mazzoni V., Cosci F., Lucchi A., Santini L. Occurrence of leafhoppers (Auchenorrhyncha, Cicadellidae) in three vineyards of the Pisa district. Proceedings of the IOBC/WPRS Bulletin.

[40] Lessio F., Tedeschi R., Pajoro M., Alma A. (2009). Seasonal progression of sex ratio and phytoplasma infection in *Scaphoideus titanus* Ball (Hemiptera: Cicadellidae). Bull. Entomol. Res..

[41] López Díez J.J. (2019). Reproductive Strategy of the Leafhopper *Orientus ishidae* Matsumura (Hemiptera: Cicadellidae). Master’s Thesis.

[42] Hunt R.E., Nault L.R. (1991). Roles of interplant movement, acoustic communication, and phototaxis in mate-location behavior of the leafhopper *Graminella Nigrifrons*. Behav. Ecol. Sociobiol..

[43] Mazzoni V., Eriksson A., Anfora G., Lucchi A., Virant-Doberlet M., Cocroft R., Gogala M., Hill P., Wessel A. (2014). Active Space and the Role of Amplitude in Plant-Borne Vibrational Communication. Studying Vibrational Communication. Animal Ssignals and Communication.

[44] Miles P.W., Treherne J.E., Berridge M.J., Wigglesworth V.B. (1972). The Saliva of Hemiptera, In Advances in Insect Physiology.

[45] Backus E.A. (1988). Sensory systems and behaviours which mediate hemipteran plant-feeding: A taxonomic overview. J. Insect Physiol..

[46] Backus E.A., Serrano M.S., Ranger C.M. (2005). Mechanisms of hopperburn: An overview of insect taxonomy, behavior, and physiology. Annu. Rev. Entomol..

[47] Backus E.A., Habibi J., Yan F., Ellersieck M. (2005). Stylet penetration by adult *Homalodisca coagulata* on grape: Electrical penetration graph waveform characterization, tissue correlation, and possible implications for transmission of *Xylella fastidiosa*. Ann. Entomol. Soc. Am..

[48] Chuche J., Sauvion N., Thiéry D. (2017). Mixed xylem and phloem sap ingestion in sheath-feeders as normal dietary behavior: Evidence from the leafhopper *Scaphoideus titanus*. J. Insect Physiol..

[49] Cornara D., Garzo E., Morente M., Moreno A., Alba-Tercedor J., Fereres A. (2018). EPG combined with micro-CT and video recording reveals new insights on the feeding behavior of *Philaenus Spumarius*. PLoS ONE.

[50] Jarausch B., Weintraub P.G. Spread of phytoplasma diseases by insect vectors: An introduction. Proceedings of the New Perspectives in Phytoplasma Disease Management.

[51] Jin S., Chen Z.M., Backus E.A., Sun X.L., Xiao B. (2012). Characterization of EPG waveforms for the tea green leafhopper, *Empoasca Vitis* Göthe (Hemiptera: Cicadellidae), on tea plants and their correlation with stylet activities. J. Insect Physiol..

[52] Miles P.W. (1999). Aphid Saliva. Biol. Rev..

[53] Nicholson S.J., Hartson S.D., Puterka G.J. (2012). Proteomic analysis of secreted saliva from russian wheat aphid (*Diuraphis noxia* Kurd.) biotypes that differ in virulence to wheat. J. Proteomics.

[54] Sharma A., Khan A.N., Subrahmanyam S., Raman A., Taylor G.S., Fletcher M.J. (2014). Salivary proteins of plant-feeding hemipteroids–implication in phytophagy. Bull. Entomol Res..

[55] Van Bel A.J., Will T. (2016). Functional evaluation of proteins in watery and gel saliva of aphids. Front. Plant. Sci..

[56] Rubia-Sanchez E., Suzuki Y., Arimura K., Miyamoto K., Matsumura M., Watanabe T. (2003). Comparing *Nilaparvata Lugens* (Stal) and *Sogatella Furcifera* (Horvath) (Homoptera: Delphacidae) feeding effects on rice plant growth processes at the vegetative stage. Crop Prot..

[57] Ab-Ghaffar M.B., Pritchard J., Ford-Lloyd B. (2011). Brown planthopper (*N. lugens* Stal) feeding behaviour on rice germplasm as an indicator of resistance. PLoS ONE.

[58] Cabauatan P.Q., Cabunagan R.C., Choi I.R., Heong K.L., Hardy B. (2009). Rice viruses transmitted by the brown planthopper *Nilaparvata lugens* Stål. Planthoppers: New Threats to the Sustainability of Intensive Rice Production Systems in Asia.

[59] Lei W., Li P., Han Y., Gong S., Yang L., Hou M. (2016). EPG Recordings reveal differential feeding behaviors in *Sogatella Furcifera* in response to plant virus infection and transmission success. Sci. Rep..

[60] Backus E.A., Shih H.T. (2020). Review of the epg waveforms of sharpshooters and spittlebugs including their biological meanings in relation to transmission of *Xylella Fastidiosa* (Xanthomonadales: Xanthomonadaceae). J. Insect Sci..

[61] Avosani S., Berardo A., Pugno N.M., Verrastro V., Mazzoni V., Cornara D. (2021). Vibrational disruption of feeding behaviors of a vector of plant pathogen. Entomol. Gen..

[62] Baric S., Öttl S., Dalla Via J. Infection rates of natural psyllid populations with ‘*Candidatus* Phytoplasma mali’in South Tyrol (Northern Italy). Proceedings of the 21st International Conference on Virus and other Graft Transmissible Diseases of Fruit Crops.

[63] Carraro L., Ferrini F., Ermacora P., Loi N., Labonne G. (2008). Infectivity of *Cacopsylla picta* (syn. *Cacopsylla costalis*), vector of “*Candidatus* Phytoplasma mali” in northeast Italy. Acta Hortic..

[64] Jarausch B., Fuchs A., Schwind N., Krczal G., Jarausch W. (2007). *Cacopsylla picta* as most important vector for ‘*Candidatus* Phytoplasma mali’ in Germany and neighbouring regions. Bull. Insectology.

[65] Malagnini V., Pedrazzoli F., Gualandri V., Zasso R., Bozza E., Fiamingo F., Pozzebon A., Mori N., Ioriatti C. (2010). Detection of “*Candidatus* Phytoplasma mali” in different populations of *Cacopsylla Melanoneura* in Italy. Bull. Insectology.

[66] Oppedisano T., Panassiti B., Pedrazzoli F., Mittelberger C., Bianchedi P.L., Angeli G., de Cristofaro A., Janik K., Anfora G., Ioriatti C. (2020). Importance of psyllids’ life stage in the epidemiology of apple proliferation phytoplasma. J. Pest Sci..

[67] Bosco D., Marzachì C., Brown J.K. (2016). Insect transmission of phytoplasmas. Vector-Mediated Transmission of Plant Pathogens.

[68] Danielli A., Bertaccini A., Vibio M., Mori N., Murari E., Posenato G., Girolami V. (1996). Detection and molecular characterization of phytoplasmas in the planthopper *Metcalfa pruinosa* (Say)(Homoptera: Flatidae). Phytopathol. Mediterr..

[69] Tedeschi R., Lauterer P., Brusetti L., Tota F., Alma A. (2009). Composition, abundance and phytoplasma infection in the hawthorn psyllid fauna of northwestern.n Italy. Eur. J. Plant Pathol..

[70] Miñarro M., Somoano A., Moreno A., García R.R. (2016). Candidate insect vectors of apple proliferation in Northwest Spain. SpringerPlus.

[71] Cainelli C., Forno F., Mattedi L., Grando M.S. Can apple aphids be vectors of “*Candidatus* Phytoplasma mali”?. Proceedings of the International Workshop on Arthropod Pest Problems in Pome Fruit Production.

[72] Mattedi L., Forno F., Cainelli C., Grando M.S., Jarausch W. (2008). Research on *Candidatus* phytoplasma mali transmission by insect vectors in Trentino. Acta Hortic..

[73] Bosco D., Tedeschi R., Dickinson M., Hodgetts J. (2013). Insect Vector Transmission Assays. Phytoplasma. Methods in Molecular Biology.

